# DEVELOPMENT OF A CULTURALLY TAILORED COST MANAGEMENT INTERVENTION INFORMED BY CAREGIVER VOICES

**DOI:** 10.1093/geroni/igad104.1999

**Published:** 2023-12-21

**Authors:** Donna Benton, Susanna Mage, Kathleen Wilber, Kylie Meyer

**Affiliations:** University of Southern California, Los Angeles, California, United States; University of Southern California, Los Angeles, California, United States; University of Southern California, Los Angeles, California, United States; Case Western Reserve University, Cleveland, Ohio, United States

## Abstract

Caregivers spend an average of 20% of their annual household income on out-of-pocket caregiving costs. Yet, no evidence-based interventions help family caregivers manage these expenses and financial stressors. Responses to financial strain must be culturally tailored to Latino family caregivers, whose out-of-pocket care costs comprise nearly half their annual household income. This presentation describes the development of the CONFIDENCE Financial Education Intervention, a 5-week psychoeducational intervention informed by the Resourcefulness and Quality of Life Theory and made to address the needs of Latino family caregivers. To ensure CONFIDENCE was responsive to the needs of Latino caregivers, the investigators 1) conducted 11 qualitative in-depth interviews with caregivers to learn about their experiences managing care costs and 2) convened a community advisory council of Latino family caregivers to guide intervention components, content, and delivery. Council recommendations guided multiple intervention modifications. For example, based on these recommendations from the council, the investigator team expanded the intervention’s target audience from focusing on women to including caregivers of all genders so families could participate together (familialism). Findings from the interviews and the council overlapped. During interviews, caregivers reported feeling worried about the growth of future caregiving costs increasing; similarly, the council described the importance of helping caregivers understand how costs would change throughout the disease trajectory. Caregiver interviews and the advisory council also emphasized the importance of addressing the needs of middle-income caregivers to prevent future financial adversity. By integrating the caregiver voices, we developed an intervention tailored to meet the needs of Latino families.

